# Phenology of marine turtle nesting revealed by statistical model of the nesting season

**DOI:** 10.1186/1472-6785-6-11

**Published:** 2006-08-31

**Authors:** Marc Girondot, Philippe Rivalan, Ronald Wongsopawiro, Jean-Paul Briane, Vincent Hulin, Stéphane Caut, Elodie Guirlet, Matthew H Godfrey

**Affiliations:** 1 Ecologie, Systématique et Evolution, UMR 8079, Université Paris-Sud, ENGREF et CNRS, Bât. 362, 91405 Orsay cedex, France; 2 Réserve Naturelle de 1'Amana, 270 avenue Paul Henri, 97319 Awala-Yalimapo, Guyane française; 3 NC Wildlife Resources Commission, 1507 Ann St., Beaufort, NC 28516, USA

## Abstract

**Background:**

Marine turtles deposit their eggs on tropical or subtropical beaches during discrete nesting seasons that span several months. The number and distribution of nests laid during a nesting season provide vital information on various aspects of marine turtle ecology and conservation.

**Results:**

In the case of leatherback sea turtles nesting in French Guiana, we developed a mathematical model to explore the phenology of their nesting season, derived from an incomplete nest count dataset. We detected 3 primary components in the nest distribution of leatherbacks: an overall shape that corresponds to the arrival and departure of leatherback females in the Guianas region, a sinusoidal pattern with a period of approximately 10 days that is related to physiological constraints of nesting female leatherbacks, and a sinusoidal pattern with a period of approximately 15 days that likely reflects the influence of spring high tides on nesting female turtles.

**Conclusion:**

The model proposed here offers a variety of uses for both marine turtles and also other taxa when individuals are observed in a particular location for only part of the year.

## Background

Many species migrate and hence are present at a particular geographic location during only part of the year [1,2]. This behavior often produces 'tidal-waves' in the density of individuals along the migration route [3] with a lower frequency signal at wintering or aestivating grounds where the animals tend to stay longer. Accurate detection and quantification of individuals of a population during migration is challenging because the movement of individuals often masks or amplifies the true size of the population, particularly when not all individuals migrate each year [4]. Periodic signals of high frequency may also be observed even when animals are resident at a particular location. For example, the presence of auklets peaks in the morning and in the evening at some colonies [5]. In this latter example, population estimates may vary depending on whether the crepuscular peaks in presence were taken into account [5]. Marine turtles exhibit variable seasonal migration patterns, with adult females being present on nesting beaches for only part of the year [6]. When on the beach, nesting sea turtles can be observed and counted as part of estimating population size [7].

Calculating the size of a marine turtle population is an essential step in assessing population status and trends [eg. [8]]. There are various challenges associated with directly counting the total number of individuals in a marine turtle population, including cryptic life history stages, trans-oceanic dispersal, and nonsequential annual reproduction [9]. As a result, researchers have traditionally relied on enumerating numbers of nests laid by a population as an index of population size [7]. Because marine turtles nest often on open sandy tropical and subtropical beaches, and because the adult females leave wide deep tracks on the beach after nesting, it is a relatively easy task to identify a sea turtle nesting crawl [10]. However, not every crawl of a female sea turtle on the beach results in a nest; for instance, when a female is disturbed before oviposition, she may abandon the nesting attempt before successfully laying eggs [10]. If tracks from non-nesting and nesting turtles are confounded during monitoring, this may strongly bias the estimated number of nests laid during a night. In the case of leatherbacks (*Dermochelys coriacea*), this potential bias can be greatly reduced by counting only those tracks that also contain a body-pit made during the nesting process: it is relatively rare (< 10% of the time) for leatherbacks to abandon the nesting process once they have made a body pit [10]. Another challenge in counting sea turtle nests is that nesting seasons usually span several months and turtles can lay their eggs on remote beaches that are difficult to access. As a result, for many sea turtle monitoring programs there are often temporal and/or spatial gaps in monitoring effort that must be corrected for, particularly when comparing datasets from different years or populations.

Several correction factors for gaps in sea turtle nest counts have been previously suggested [11-13]. One simple method used to correct for an incomplete dataset from a sea turtle nesting beach is n^
 MathType@MTEF@5@5@+=feaafiart1ev1aaatCvAUfKttLearuWrP9MDH5MBPbIqV92AaeXatLxBI9gBaebbnrfifHhDYfgasaacH8akY=wiFfYdH8Gipec8Eeeu0xXdbba9frFj0=OqFfea0dXdd9vqai=hGuQ8kuc9pgc9s8qqaq=dirpe0xb9q8qiLsFr0=vr0=vr0dc8meaabaqaciaacaGaaeqabaqabeGadaaakeaacuWGUbGBgaqcaaaa@2E21@ = *n*/(*proportion of beach covered*) [adapted from [30]]. This correction assumes that all beach sections being monitored are homogeneous; a bias is introduced if they are not. This may be irrelevant if the introduced bias is constant and one is interested only in trends of the abundance of adult females [14]. However, if the sections of the beach surveyed are not similar from year to year, the introduced bias will fluctuate, rendering this simple correction factor unsuitable. A more complicated situation occurs when there are temporal gaps during monitoring of the nesting season. As the temporal distribution of nests during a season tends to be bell-shaped, a simple extrapolation based on the proportion of the season that was monitored to correct for temporal gaps in nest monitoring will likely give false results. Furthermore, the distribution of nests during the season is dependent on many factors, such as the total number of nesting females, the timing of initial nesting dates for individual females and the distribution of total number of nests laid during the season by each female. Marine turtles are iteroparous [15], nesting up to 14 times in a single season in the case of leatherbacks in French Guiana [16]. The internesting interval for females varies according to species: from 10 days on average for leatherbacks [16] up to 28 days for olive ridley (*Lepidochelys olivacea*) females in mass nesting events [17]. The internesting interval within some species can vary with fluctuating water temperatures [18].

The temporal pattern of internesting intervals within a single nesting season can produce fluctuations in the total distribution of nests, resulting in successive peaks of nests on certain days during the nesting season. Peaks in nesting activity every ~15 days in apparent synchrony with the lunar phase and tide level have been observed on the leatherback nesting beach of Ya:lima:po beach in French Guiana [16]. This synchrony may be due to some females adjusting their subsequent nesting date to coincide with a particular lunar phase/tide level; nevertheless, the overall mean internesting interval is 10 days for this population [16].

The objective of this study was to develop a method that can transform an incomplete dataset of nesting activity for a sea turtle population into a dataset that accurately describes both the pattern of nesting distribution during the nesting season and any possible short-term fluctuations. The gaps in the data could be both spatial and temporal. The objective was achieved by both extrapolation and interpolation, based on a derived realistic model of the nesting season that included parameters that account for variation in the shape of the nesting season. The resultant model is useful not only for calculating the total number of nests laid (and its standard error) during an entire season, but also for facilitating comparisons across nesting beach datasets that contain dissimilar spatio-temporal gaps.

**Figure 1:**
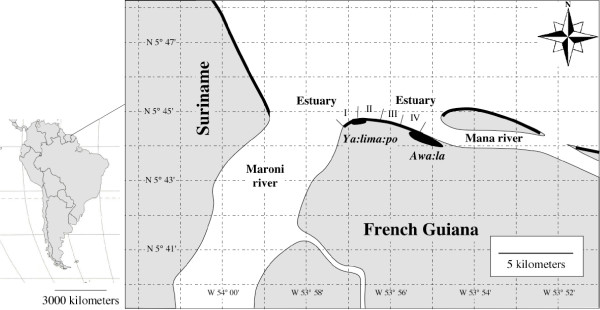
Map of Ya:lima:po beach within the estuary of the Maroni and Mana rivers. Nesting beach sections used in this study are indicated in bold.

We tested the developed method using an incomplete leatherback nest count dataset (2002) on Ya:lima:po nesting beach in French Guiana (Figure 1), and tested it using a complete nest count dataset from 1987.

## Results

### Ya:lima:po entire beach – 2002 data (Table 1)

A total of 3356 nests were counted during 171 field days from 1/3/2002 until 31/12/2002 on the Ya:lima:po nesting beach. The Akaike Information Content (AIC) of the 8 models describing the shape of the nesting season was lowest when the *K*_1 _parameter was fixed to 0. However, the various model combinations with *K*_1 _= 0 and/or *K*_2 _= 0 had essentially the same support (i.e. Akaike weights were similar). In contrast, the models with the *min *parameter fixed to 0 showed considerable lower support. As outlined above, our initial model selection criteria were a) low AIC value and b) fewer parameters. From this, the initial model we retained used 9 parameters (*S*_1_, *P*_1_, *S*_2_, *P*_2_, *Min*, *Max*, *a*, *b*, *c*). The goodness of fit for this model was r^2 ^= 0.85 and it estimated a seasonal total of 4652 ± 73SD nests.

**Figure 2:**
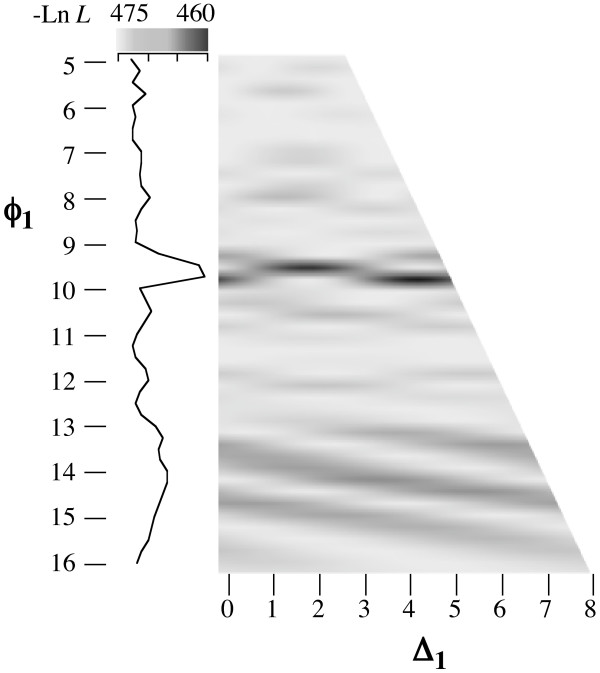
Map of-Ln Likelihood with varying φ_1 _and Δ_1 _parameters for 2002 nesting season.

We added a sinusoid function to this pattern, by generating a map of -Ln Likelihood with varying φ_1 _and Δ_1 _parameters (Figure 2). The maximum likelihood was observed for φ_1 _= 9.5 and Δ_1 _= 6.75. These values were used as initial points to search for values of α_1_,β_1_, φ_1_, Δ_1_, and τ_1 _parameters that also maximized likelihood. We then searched for a simpler model assuming α_1 _= 0 and/or β_1 _= 0 and/or τ_1 _= l. The model with lowest AIC had a fitted value of τ_1_. However, based on Akaike weights, this model exhibited a goodness of fit similar to the model with τ_1 _= 0 (0.50 *vs*. 0.49). As this latter model was simpler, we retained it for further tests. When one sinusoid was added, the AIC = 940.65, much lower than AIC = 963.22 prior to the addition of the sinusoid. The goodness of fit for this model was r^2 ^= 0.87 and the total number of nests is estimated to be 4719 ± 62SD.

**Figure 3:**
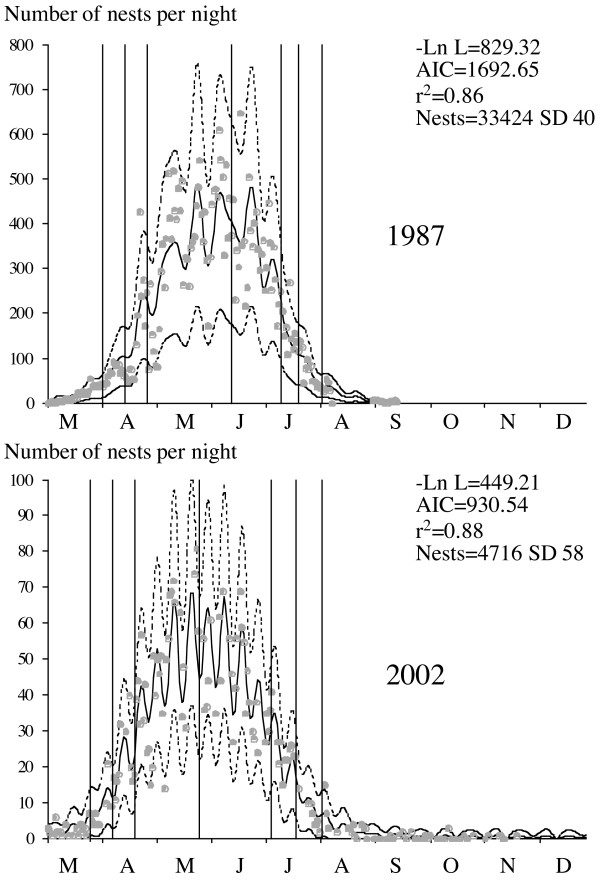
Leatherback nest distribution during the 1987 and 2002 nesting seasons in Ya:lima:po nesting beach. Black points are the nest counts. The central curve is the best fitted model and the upper and lower curves are the error envelopes (±2 SD). The vertical bars represent, from right to left, d+10%, d+25%, d+50%, day at peak, d-50%, d-25%, d-10% values (see text). -Ln L is -logarithm of maximum likelihood for the corresponding model and AIC is the Akaike Information Content.

A second sinusoid was added using the same procedure. The map of -Ln Likelihood showed a minimum for (φ_1 _= 14 and Δ_1 _= 4. These values were used as initial points to search for values of α_2_, β_2_, φ_2_, Δ_2_, and τ_2 _parameters that maximized likelihood. Then, we searched for a simpler model by assuming α_2 _= 0 and/or β_2 _= 0 and/or τ_2 _= l. The model with the lowest AIC value assumed that τ_2 _= l. When the second sinusoid was added, the AIC = 930.54 and was lower than the AIC obtained with one sinusoid (AIC = 940.65). The goodness of fit for this two-sinusoid model was r^2 ^= 0.88 and the total number of nests it estimated was 4716 ± 58SD (Figure 3). The addition of a third sinusoid gave the model lower a AIC (923.90), but based on Akaike weight the overall fit was not significantly better than the model with only two sinusoids. Therefore, the best-fit model based on both AIC and Akaike weight criteria had two sinusoids.

### Analysis of beach sections – 2002 data

Nest distribution for each beach section (Figure 1) was fitted as previously described. A homogeneity test showed that the global shape of nesting was significantly different for the four beach sections (LRT = 391.65, DF = 18, p < 0.0001). A similar conclusion was obtained when only the beginning of the nesting season was assumed to be identical (LRT = 67.23, DF = 9, p < 0.0001) or the end of nesting season was assumed to be identical (LRT = 294.27, DF = 9, p < 0.0001). The index d+10% that describes the beginning of the nesting season showed a significant spatial tendency, with the nesting season beginning later in the western section of the beach (Kendall rank correlation, p = 0.04), but no spatial influence was observed for either the peak of the season or the d-10% descriptor of the end of the nesting season (p > 0.05).

**Figure 4:**
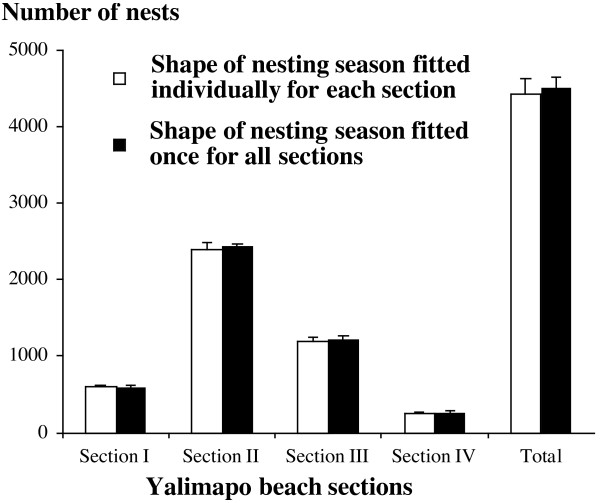
Estimated nest numbers for various beach sections if each beach section was fitted using its own model (4 different models, open bars) or if a single fitted model was used for all beach sections (1 single model, closed bars).

Although the shapes of nest distribution among the different beach sections were significantly different, the use of a single model for the global shape of nest distribution during the nesting season did not affect the estimates of total nest numbers for each zone (Figure 4).

**Figure 5:**
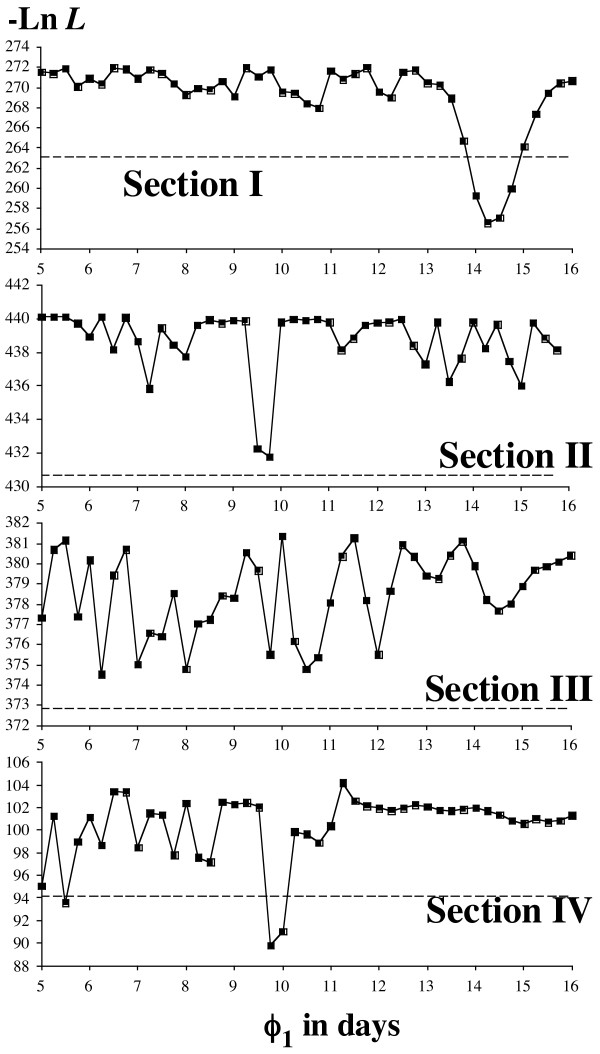
Likelihood for nest distributions on the different beach sections when one sinusoid with varying period was added. The dashed line defined the limit of -Ln *L *statistical significance using Akaike weight after applying a Bonferronni correction for multiple tests.

When one sinusoid with a period varying from 5 to 16 days by steps of 0.25 days was added to the models, the beach sections also showed strong differences. Indeed, the sinusoidal period appeared to be different according to the position of the beach section analyzed. The 14.25-days signal was significantly strong within section I whereas the 9.8-days signal was significantly strong within section IV (Figure 5).

### Ya:lima:po entire beach – 1987 data (Table 2)

A total of 33337 nests were counted during 178 field days from 1/3/1987 until 15/9/1987 on Ya:lima:po nesting beach. Only 22 daily counts were missing at the end of the nesting season. This time-series can therefore be considered as essentially complete as the nesting after July is relatively sparse. The AIC of the 8 models describing the overall shape of nesting season was lowest when the *K*_1 _parameter was fixed to 0, but was not significantly different from several other models based on Akaike weight (Table 2A). We choose the first one in the series with Akaike weight > 0.05 and minimum number of fitted parameters.

We added a sinusoid function to this pattern, by generating a map of -Ln Likelihood with varying φ_1 _and Δ_1 _parameters. The maximum likelihood was observed for (φ_1 _= 15.25 and Δ_1 _= ll. These values were used as initial points to search for values of the α_1_, β_1_, φ_1_, Δ_1 _and τ_1 _parameters that maximized likelihood. We then searched for a simpler model assuming α_1 _= 0 and/or β_1 _= 0 and/or τ_1 _= 1 (Table 2A). The model with the lowest AIC had a nearly identical AIC value to the model that was had one less parameter (τ_1 _was set to 1). Using the principle of parsimony, we retained the model with fewer parameters for further exploration. Second and third sinusoidal signals were added to the model; we selected the single model within all groups of models (0, 1, 2, or 3 sinusoidal signals added) with the lowest AIC values. Of these four, we found that the model with 2 sinusoidal signals had the lowest Akaike weight (Table 2). The two sinusoidal periods fitted were 10.60 and 15.34 days. The Gratiot et al. model [19] when applied to this time-series was strongly rejected compared to the model presented here (AIC 2047.30 *vs*. 1693.67; p < 10^-10 ^based on Akaike weight). The number of observed nests during the 178 night patrols was 33337. The number of nests calculated by the models was as follows: 33223 (ε = -0.34%) for no sinusoidal signal, 33305 (ε = -0.09%) for 1 sinusoidal signal added, and 33118 (ε = -0.65%) for 2 sinusoidal signals added (Figure 3). All three estimates are close to the observed number of nests (note that the number of nests reported in Table 2 are for the entire nesting season, including the final 22 days without available nest counts).

The periodic signal within number of nests that was detected in the 1987 database comprised of 161 consecutive day counts can be also tested using classical mathematical tools. The Multi-Taper method (MTM) of spectral analysis provides a means for spectral estimation [20,21] of a time series that is believed to exhibit a spectrum containing both continuous and singular components. A MTM test for white noise in this time-series indeed detects a significant period signal at both 10.60 days (p < 0.01) and at 15.34 days (p < 0.05). However, the MTM method can be used only with continuous time-series, which therefore makes it ineffective when analyzing marine turtle datasets that contain gaps in coverage.

## Discussion

The total number of nests deposited during the nesting season is often used as an index of abundance for marine turtle species. To obtain reliable data, monitoring of the nesting beach should be conducted on a regular basis during the nesting season. However, nesting seasons can span up 6 months or more, making it challenging to effectively collect data on a daily basis [12]. It is common for nesting beach datasets to be missing information, either during, at the beginning and/or at end of the nesting season. One means to account for data gaps during the nesting season is to employ interpolation. For instance, Girondot & Fretey [16] were able to fill in data gaps for a leatherback nesting population using interpolation with a small associated error (5%). However, this procedure is inadequate if the beginning and/or the end of a nesting season is not monitored. Troëng *et al*. [8] used a moving average, but again, the behavior of such a function can be erratic at the boundaries of the time-series. Yet gaps at the beginning or end of the season are common because research teams tend to concentrate monitoring effort at the peak of the nesting season. To be able to extrapolate data, one needs an equation that accurately describes the global nesting season. A recently proposed symmetrical model of a leatherback nesting season [19] is inadequate for modeling the dataset from Ya:lima:po because the time series analyzed above was not symmetrical (for example, based on the 2002 time-series: symmetric model with S_1 _= S_2 _and (K_1 _= K_2_) ≠ 0 AIC 966.26 or S_1 _= S_2 _and K_1 _= K_2 _= 0 AIC 964.51 vs. asymmetrical model – see Table 1B – S_1 _≠ S_2 _and K_1 _= K_2 _= 0 AIC 963.22). Also, nest counts outside the main sinusoid were calculated separately before the fitting procedure used by Gratiot *et al*. [19]. Finally, a fitting procedure based on two rounds should be avoided, as they generally do not converge on the best fit of the data.

In contrast, we have shown that the best fit model describing the nesting season of leatherback at Ya:lima:po exhibits three components: an overall shape describing the nesting season and two sinusoids signals. For 1987, one signal has a period of 10.60 ± 0.0003 SD days and the other a period of 15.34 ± 0.0004 SD days. For 2002, one signal has a period of 9.74 ± 0.01 SD days and the other a period of 14.11 ± 0.004 SD days. The first signal likely represents the reproductive cycle of females that nest sequentially during the nesting season, with nests separated by approximately nine to ten days [16]. The second signal could be related to the lunar or tidal phase when full and new moon alternate every 14.25 days [16]. This signal was particularly strong for the beach section located furthest within the estuary (Section I, Figure 5). In this section, the peak of nesting coincided with the spring high tides observed during each full and new moons. Interestingly, it has been observed that greater numbers of female leatherbacks become trapped in exposed mud flats after nesting during neap tides on the nearby beaches in Suriname [22]. However, we do not think that the peak of nesting during spring tides represents a selective avoidance response to this potential mortality factor, for the following two reasons. First, the actual mortality rate is low for females that are temporarily trapped in the mud-flats (M.G., personal observation), making it unlikely that this would constitute a strong selective pressure. Second, the peak of nesting during spring tide is not observed in all beach sections (Figure 4). Nesting at high tide during spring tide could be also viewed as a way to minimize energy expenditure while selecting a suitable site for nesting on the beach [23]. Alternatively, the peaks in nesting distribution at spring high tide could result from currents in the Maroni river that may prevent individual leatherbacks from nesting too far inside the estuary at neap tide (Figure 1). However, an expected complementary peak at neap tide in other beach sections further away from the Maroni was not detected (Figure 5). A third possible explanation is that a shift of female turtles from other nesting beaches in the region (Figure 1) to Ya:lima:po beach may be associated with the spring high tide. Current data are insufficient to test this hypothesis.

In French Guiana, leatherback females can adjust their return nesting date from 6 to 16 days between two nesting events in order to be closer to a full or new moon [16]. This effect was not observed in leatherbacks nesting on the Pacific coast of Costa Rica [24]. However, the number of observations available from Costa Rica was only one tenth of the number in Girondot & Fretey [16], which is insufficient to detect an effect similar to the one observed in Ya:lima:po (M.G., unpublished results). Note that even with this significant peak of nesting at the spring high tide, leatherbacks in French Guiana have a stronger signature at 9.8 days that is the characteristic nesting interval for this species [25]. Stronger and more significant periodic signals were detected at both ends of the Ya:lima:po nesting beach (Sections I and IV, Figure 5). The lack of significant signal in sections II and III is probably the consequence of reduced environmental constraints from tide or river currents for the central sections of the beach.

**Figure 6:**
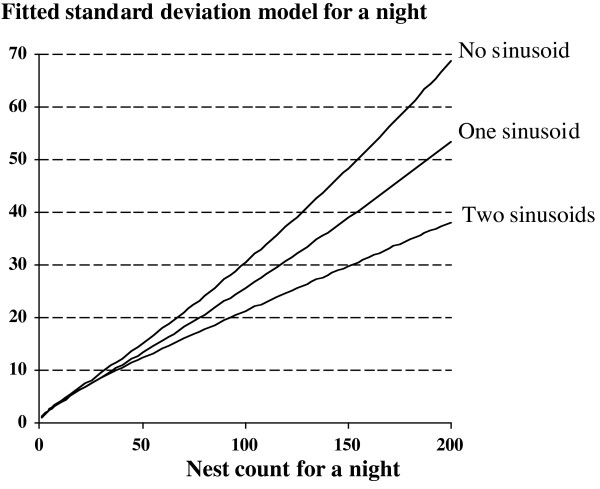
Standard deviations of the fitted model with zero, one or two sinusoids added to the global pattern.

The numbers of total nests calculated by the various methods proposed here were not significantly different: the additions of one or two sinusoidal signals in 2002 changed the estimation of total nest number from 4652 ± 73 SD (no sinusoids), to 4719 ± 62 SD (one sinusoid) and 4716 ± 58 SD (two sinusoids). Given the consistency of total nest estimates, one could question why a more complex model is needed. It should be noted that the standard deviations of the estimates were lower with the addition of sinusoids because these sinusoids explained some of the variation that was expressed by the standard deviation of the simpler models (Figure 6). Therefore, the beta risk was reduced when the estimate was derived with the more complex model.

## Conclusion

The model proposed here offers a variety of uses beyond its application in accurately estimating total counts from an incomplete dataset. It also can detect intraseasonal concentrations of animals that may be the result of behavioral interactions, synchronization with external factors and/or biological constraints. When applied to leatherbacks nesting in French Guiana, the model detected 3 principal components that contribute to the seasonal nest distribution: a global shape that corresponds to the arrival and departure of leatherback females in the Guianas region, a sinusoidal pattern with a period of approximately 10 days that is related to physiological constraints of nesting leatherbacks, and a sinusoidal pattern with a longer period that likely reflects the influence of the spring high tide on nesting turtles.

The proposed model is an improvement over previously published methods [8,16,19] but we should keep in mind that nest number is only an index of the population size. Indeed, variable remigration interval [26] and variable number of nest per female [15] affect also strongly the observed number of nests for a particular year.

## Methods

### Nesting data

Nest count data were collected in 1987 and 2002 on Ya:lima:po nesting beach in French Guiana (Figure 1). This nesting beach is approximately 4 km long and was divided into 4 sections that were roughly equal in distance. Nesting activity of leatherback turtles was monitored either by conducting daily dawn patrols to count all nest tracks made the previous night and/or by counting individual nesting females at night when total nests per night > 50 [see complete description in [27]]. False crawls are relatively rare in leatherbacks nesting in French Guiana and only tracks that reach the upper part of the beach and had signs of post-oviposition camouflaging by the female turtle were counted as nests. Nest counts were made during the primary nesting season, from 1st March (defined as day 0) until 15 September 1987 or 31 December 2002. Data for January and February were not included because leatherbacks display a smaller secondary nesting season during these months [28]. In 1987,178 nights from 1/3/1987 until 15/9/1987 were worked on the Ya:lima:po nesting beach. Only 22 daily counts were missing at the end of the nesting season (10/8/1987 to 31/8/1987). This time-series can be considered as nearly complete as the nesting after July is relatively rare. A total of 33337 nests were counted during the 1987 season. Of the 305 days of the 2002 primary nesting season, 3356 nests were counted on 171 nights (56% coverage). Whether observed at night or the following morning, each nest was assigned the date prior to midnight of the night it was laid. The lunar phase for each night was also calculated using internet moon phase calculator [29].

### The model to describe the nesting season

Nesting seasons of marine turtles are typically characterized by a peak of nesting approximately during the middle of the nesting season. The number of nests at the start and end of the nesting season is low ; generally less than one nest per week or per month in some populations (unpublished data). This typical pattern has been modeled as a sinusoid function [19]. However, this function presupposes that the shape of the seasonal nesting distribution is symmetrical. Several different populations of marine turtles display asymmetrical peaked patterns associated with their nesting season [e.g. [30-32]]. Moreover, the assumption that the temporal nesting distribution of sea turtles is a simple sinusoid has several inherent statistical weaknesses (see below).

As an alternative, the seasonal nesting pattern can be modeled using the product of two sigmoid equations, the first one ranging from 0 to 1 and the second one from 1 to 0. The product shows a 0-1-0 pattern if the transition of the first equation is observed at an abscissa of lower value than the second one. For each sigmoid equation, we use a modified form of the Verhulst equation [33] that allows asymmetry to be set. Note that the first-order derivative of this equation is similar to the Richards equation [34]:

M(d)=(1+(2eK−1)e(1S(P−d)))−1/eK     (1)
 MathType@MTEF@5@5@+=feaafiart1ev1aaatCvAUfKttLearuWrP9MDH5MBPbIqV92AaeXatLxBI9gBaebbnrfifHhDYfgasaacH8akY=wiFfYdH8Gipec8Eeeu0xXdbba9frFj0=OqFfea0dXdd9vqai=hGuQ8kuc9pgc9s8qqaq=dirpe0xb9q8qiLsFr0=vr0=vr0dc8meaabaqaciaacaGaaeqabaqabeGadaaakeaacqWGnbqtcqGGOaakcqWGKbazcqGGPaqkcqGH9aqpdaqadiqaaiabigdaXiabgUcaRmaabmGabaGaeGOmaiZaaWbaaSqabeaacqWGLbqzdaahaaadbeqaaiabdUealbaaaaGccqGHsislcqaIXaqmaiaawIcacaGLPaaacqWGLbqzdaahaaWcbeqaamaabmGabaWaaSaaaeaacqaIXaqmaeaacqWGtbWuaaGaeiikaGIaemiuaaLaeyOeI0IaemizaqMaeiykaKcacaGLOaGaayzkaaaaaaGccaGLOaGaayzkaaWaaWbaaSqabeaacqGHsislcqaIXaqmcqGGVaWlcqWGLbqzdaahaaadbeqaaiabdUealbaaaaGccaWLjaGaaCzcamaabmGabaGaeGymaedacaGLOaGaayzkaaaaaa@501C@

where *d *is the Julian date, *P *is related to the dates before and after the peak nesting day when there is an observed maximum rate of change (increase or decrease) in nest numbers, and *S *and *K *are related to the change in slope of the change in nest numbers at date *P*.

The value of *M*(*d*) ranges from 0 to 1 with *M*(*d*) = 0.5 for *P *= *d*, with *d *being the number of days since the start of the nesting season. The steepness of *M*(*d*) at *P *= *d *depends on *S *and *K *values. *M*(*d*) is increasing when *S *is negative (*i.e*. at the start of the nesting season) and decreasing when *S *is positive (i.e. at the end of nesting season). Asymmetry around *P *is determined by a positive or negative value of *K*. The equation (1) is reduced to a simple logistic equation (i.e. symmetrical around *P*) when *K *= 0.

To avoid a computing overflow, the equation (1) was simplified to

M(d)=e−1/eK((2eK−1)e(1S(P−d)))
 MathType@MTEF@5@5@+=feaafiart1ev1aaatCvAUfKttLearuWrP9MDH5MBPbIqV92AaeXatLxBI9gBaebbnrfifHhDYfgasaacH8akY=wiFfYdH8Gipec8Eeeu0xXdbba9frFj0=OqFfea0dXdd9vqai=hGuQ8kuc9pgc9s8qqaq=dirpe0xb9q8qiLsFr0=vr0=vr0dc8meaabaqaciaacaGaaeqabaqabeGadaaakeaacqWGnbqtcqGGOaakcqWGKbazcqGGPaqkcqGH9aqpcqWGLbqzdaahaaWcbeqaaiabgkHiTiabigdaXiabc+caViabdwgaLnaaCaaameqabaGaem4saSeaaSWaaeWaceaadaqadiqaaiabikdaYmaaCaaameqabaGaemyzau2aaWbaaeqabaGaem4saSeaaaaaliabgkHiTiabigdaXaGaayjkaiaawMcaaiabdwgaLnaaCaaameqabaWaaeWaceaadaWcaaqaaiabigdaXaqaaiabdofatbaacqGGOaakcqWGqbaucqGHsislcqWGKbazcqGGPaqkaiaawIcacaGLPaaaaaaaliaawIcacaGLPaaaaaaaaa@4BD7@ when (2eK−1)e(1S(P−d))
 MathType@MTEF@5@5@+=feaafiart1ev1aaatCvAUfKttLearuWrP9MDH5MBPbIqV92AaeXatLxBI9gBaebbnrfifHhDYfgasaacH8akY=wiFfYdH8Gipec8Eeeu0xXdbba9frFj0=OqFfea0dXdd9vqai=hGuQ8kuc9pgc9s8qqaq=dirpe0xb9q8qiLsFr0=vr0=vr0dc8meaabaqaciaacaGaaeqabaqabeGadaaakeaadaqadiqaaiabikdaYmaaCaaaleqabaGaemyzau2aaWbaaWqabeaacqWGlbWsaaaaaOGaeyOeI0IaeGymaedacaGLOaGaayzkaaGaemyzau2aaWbaaSqabeaadaqadiqaamaalaaabaGaeGymaedabaGaem4uamfaaiabcIcaOiabdcfaqjabgkHiTiabdsgaKjabcMcaPaGaayjkaiaawMcaaaaaaaa@3E30@ and to 12e(d−PSeK)
 MathType@MTEF@5@5@+=feaafiart1ev1aaatCvAUfKttLearuWrP9MDH5MBPbIqV92AaeXatLxBI9gBaebbnrfifHhDYfgasaacH8akY=wiFfYdH8Gipec8Eeeu0xXdbba9frFj0=OqFfea0dXdd9vqai=hGuQ8kuc9pgc9s8qqaq=dirpe0xb9q8qiLsFr0=vr0=vr0dc8meaabaqaciaacaGaaeqabaqabeGadaaakeaadaWcaaqaaiabigdaXaqaaiabikdaYaaacqWGLbqzdaahaaWcbeqaamaabmGabaWaaSaaaeaacqWGKbazcqGHsislcqWGqbauaeaacqWGtbWucqWGLbqzdaahaaadbeqaaiabdUealbaaaaaaliaawIcacaGLPaaaaaaaaa@38FA@ when *K *> 3 and sign(*P*-*d*) = sign(*S*).

The mathematical description of a nesting season can therefore be expressed as:

*N*(*d*) = *min *+ (*max *- *min*) · (*M*_1 _(*d*) · *M*_2 _(*d*))       (2)

with *M*_1_(*d*) and *M*_2_(*d*) being the first and second halves of the nesting season, respectively. The difference between the two largely rests on the sign of the *S *parameter: *S*_1 _is negative, *S*_2 _is positive and *P*_1 _<*P*_2_. The parameter *min *is the basal level of nesting outside the nesting season and *max-min *is a scaling factor. Note that *max *is not the maximum of the function because (*M*_1_(*d*) · *M*_2 _(*d*)) can be lower than 1 at the peak of nesting season. The maximum must be calculated numerically.

The entire nesting season can be expressed using equation (2), which is based on 8 parameters. The number of parameters can be reduced using the Verhulst equation around *P*_1 _(*K*_1 _= 0), *P*_2 _(*K*_2 _= 0), or both. The basal level of nests *min *can be fixed to 0. Also, if it is assumed that the beginning and the end of nesting season show similar shapes, this can be expressed by setting *S*_1 _= -*S*_2 _and *K*_1 _= *K*_2_. The most reduced form of equation 2 uses only 4 parameters (*min *= 0, *K*_1 _= *K*_2 _= 0, *S*_1 _= -*S*_2_). The test for an asymmetrical shape of the nesting season was performed by forcing S_2 _= -S_1 _and K_1 _= K_2_.

Equation (2) describes the global shape of the nesting season. A more complete model could also incorporate periodic variation within the nesting season. Traditionally, spectral analysis has been used to describe periodic patterns, but the discontinuities in the daily nest count dataset makes the use of these tools inappropriate [35]. Instead, a sinusoid function was incorporated into the equation (2) and the corresponding parameters are fitted against the daily nest counts. When several periodic signals were detected, the sum of/sinusoid equations was used:

N′(d)=N(d)+∑k=1l(sin⁡(2πd+Δkφk)(αk+βk⋅N(d)τk))     (3)
 MathType@MTEF@5@5@+=feaafiart1ev1aaatCvAUfKttLearuWrP9MDH5MBPbIqV92AaeXatLxBI9gBaebbnrfifHhDYfgasaacH8akY=wiFfYdH8Gipec8Eeeu0xXdbba9frFj0=OqFfea0dXdd9vqai=hGuQ8kuc9pgc9s8qqaq=dirpe0xb9q8qiLsFr0=vr0=vr0dc8meaabaqaciaacaGaaeqabaqabeGadaaakeaacuWGobGtgaqbaiabcIcaOiabdsgaKjabcMcaPiabg2da9iabd6eaojabcIcaOiabdsgaKjabcMcaPiabgUcaRmaaqahabaWaaeWaceaacyGGZbWCcqGGPbqAcqGGUbGBdaqadiqaaiabikdaYGGaciab=b8aWnaalaaabaGaemizaqMaey4kaSIaeuiLdq0aaSbaaSqaaiabdUgaRbqabaaakeaacqWFgpGzdaWgaaWcbaGaem4AaSgabeaaaaaakiaawIcacaGLPaaadaqadiqaaiab=f7aHnaaBaaaleaacqWGRbWAaeqaaOGaey4kaSIae8NSdi2aaSbaaSqaaiabdUgaRbqabaGccqGHflY1cqWGobGtcqGGOaakcqWGKbazcqGGPaqkdaahaaWcbeqaaiab=r8a0naaBaaameaacqWGRbWAaeqaaaaaaOGaayjkaiaawMcaaaGaayjkaiaawMcaaaWcbaGaem4AaSMaeyypa0JaeGymaedabaGaemiBaWganiabggHiLdGccaWLjaGaaCzcamaabmGabaGaeG4mamdacaGLOaGaayzkaaaaaa@66F8@

Parameter φ defines the period of the sinusoidal pattern and Δ is the phase shift. The amplitude of variation is governed by α, β and τ parameters. If the amplitude is independent of the number of nests, then α ≠ 0 and β = 0 (the parameter τ is not used and set to 1). If α ≠ 0 and β ≠ 0, then the amplitude(s) of the fluctuations have two components, one dependent and one independent of the number of nests. The parameter τ ≠ l renders a non-linear dependent relationship between amplitude of the fluctuations and number of nests. Equation (3) implies that sinusoid signals are additive. It can be changed to allow a multiplicative effect but this kind of model performs less well than the additive model and will not be discussed further (data not shown). The model defined with equation (3) uses 5*l *parameters more than the model defined with equation (2). Equation (3) can be simplified setting α = 0 or β = 0 and/or τ = l (3 or 4 parameters).

The curve was fitted to experimental data by maximum likelihood method using a simplex search [36] followed by a gradient search [37]. For this purpose, we assumed that the nest distribution for day *d *is normally distributed with a standard deviation σ_*a *_= Exp(*a*.*N*'(*d*)^*c *^+ *b*) where *a*, *b *and *c *are parameters that are also fitted. This function has the advantage of being strictly positive and monotonically increasing according to *N*'(*d*) when *a *and *c *are positive. It also accounts for the observed heteroskedasticity, *i.e*. nest counts were more dispersed at the peak of nesting season. An alternative is to use the lognormal distribution for nest number which has the advantage of being always positive but also requires a forced origin that is not zero as zero nests is not a possible output.

For ease of use, this modeling application has been scripted for both MacOS and Microsoft Windows platforms [38].

### Model selection and goodness of fit

Model selection was performed using the Akaike Information Content [39]. This is a ranking measure that takes into account the quality of the fit of the model to the data while penalizing for the number of parameters used:

AIC = -2 In *L *+ 2 M       (4)

where *L *= maximum likelihood, and M = the number of parameters. The models with the lowest values of AIC were retained as good candidate models and Δ_AIC _was calculated as the difference in value of AIC between a particular model and the one with the lowest AIC. Akaike weights (*w*_*i *_= exp(-Δ_AIC_/2) normalized to 1) were used to evaluate the relative support of various tested models [40]. Akaike weights can be directly interpreted as conditional probabilities for each model. Ideally, the model with the lowest AIC was kept for further testing. When two or more models possessed similar Akaike weights, the model with the lowest number of parameters was selected. When several of these models had the same number of parameters, the model with the lowest AIC among them was selected.

When varying the period of sinusoid signal within the nesting season, the significance levels of the models with or without sinusoidal signals were tested using Akaike weight after a Bonferronni correction. Let *L*_0 _be the -Ln likelihood of the best model without a sinusoid and let *B *be a matrix of *b *-Ln likelihoods (*b *is the Bonferronni correction factor) obtained by varying φ_1 _parameter and fitting β_1 _and Δ_1_. A likelihood *i *of the *B *matrix was considered as being significantly better than L_0 _if *w*_*i *_< 0.05/*b*. Therefore, a model with a sinusoid was significantly better (p < 0.05) than the model without one if the difference between likelihoods was higher than 2+ln⁡(0.05/b1−(0.05/b))
 MathType@MTEF@5@5@+=feaafiart1ev1aaatCvAUfKttLearuWrP9MDH5MBPbIqV92AaeXatLxBI9gBaebbnrfifHhDYfgasaacH8akY=wiFfYdH8Gipec8Eeeu0xXdbba9frFj0=OqFfea0dXdd9vqai=hGuQ8kuc9pgc9s8qqaq=dirpe0xb9q8qiLsFr0=vr0=vr0dc8meaabaqaciaacaGaaeqabaqabeGadaaakeaacqaIYaGmcqGHRaWkcyGGSbaBcqGGUbGBdaqadiqaamaalaaabaGaeGimaaJaeiOla4IaeGimaaJaeGynauJaei4la8IaemOyaigabaGaeGymaeJaeyOeI0YaaeWaceaacqaIWaamcqGGUaGlcqaIWaamcqaI1aqncqGGVaWlcqWGIbGyaiaawIcacaGLPaaaaaaacaGLOaGaayzkaaaaaa@421F@.

Goodness of fit was evaluated using the coefficient of determination (r^2^) between the observed and estimated nest numbers to facilitate comparisons with a previously published method [19]. However, it is important to note that the r^2 ^statistic is inadequate for non-stationary time-series such as those analyzed here, as any distribution showing a peak at the middle of the time-series will generate an inflated r^2 ^value.

For the model being fitted, the error ε in per cent on the estimate of the total number of nests was calculated as [19]:

ε=(∑t=1365N′(t)−∑t=1365n(t))/∑t=1365n(t)
 MathType@MTEF@5@5@+=feaafiart1ev1aaatCvAUfKttLearuWrP9MDH5MBPbIqV92AaeXatLxBI9gBaebbnrfifHhDYfgasaacH8akY=wiFfYdH8Gipec8Eeeu0xXdbba9frFj0=OqFfea0dXdd9vqai=hGuQ8kuc9pgc9s8qqaq=dirpe0xb9q8qiLsFr0=vr0=vr0dc8meaabaqaciaacaGaaeqabaqabeGadaaakeaadaWcgaqaaGGaciab=v7aLjabg2da9maabmGabaWaaabCaeaacuWGobGtgaqbaiabcIcaOiabdsha0jabcMcaPiabgkHiTmaaqahabaGaemOBa4MaeiikaGIaemiDaqNaeiykaKcaleaacqWG0baDcqGH9aqpcqaIXaqmaeaacqaIZaWmcqaI2aGncqaI1aqna0GaeyyeIuoaaSqaaiabdsha0jabg2da9iabigdaXaqaaiabiodaZiabiAda2iabiwda1aqdcqGHris5aaGccaGLOaGaayzkaaaabaWaaabCaeaacqWGUbGBcqGGOaakcqWG0baDcqGGPaqkaSqaaiabdsha0jabg2da9iabigdaXaqaaiabiodaZiabiAda2iabiwda1aqdcqGHris5aaaaaaa@5909@ with *n*(*t*) being the observed number of nests for night *t*.

Note that this formula assumes that nest counts are known for each day of the time series, which is rarely the case and only days with known nest counts were used to calculate ε.

The standard error for the parameters was estimated using a standard procedure to estimate the variance of a function about a point [41]. In short, when applied to the error in the location of one point this procedure is directly related to the uncertainty in the model relative to the position of the peak (mathematically represented by the standard error in the estimation of the point where the first derivative is zero). It is inversely related to the value of the second derivative about the maximum. It is approximated using a fourth order polynomial function around the fitted value for each parameter [41].

### Synthetic parameters

Not all parameters involved in equation (2) could be directly derived to describe aspects of nesting season (beginning, end, or length) because of complex interactions between them. Rather, several new statistics were calculated. The d+x% and d-x% values are the days where *N*(*d*) is equal to x% of the maximum nest number (d+ indicates the start of nesting season and d- the end of nesting season with x = 10, 25 and 50%). The values d+x% and d-x% are an index that is proportional to the beginning and the end of nesting season. The value d-x% – d+x% is an index related to the length of the nesting season. Equation (2) cannot be transformed to directly obtain the d ± x% values, rather a numerical approximation was required. The standard errors for the d ± x% values were obtained by 100 replications of equation (2) with parameter values obtained randomly from values that maximized the likelihood while taking into account the standard error. The total number of nests deposited during the entire season was the sum of the number of nests laid per night.

### Homogeneity test

The test for homogeneity of the nest distribution for several nesting beaches was performed using likelihood ratio test and AIC comparison. We assumed that L_i _was the maximum likelihood and M_i _was the number of parameters for beach section *i*. Under the hypothesis that nest distribution was the same for all these nesting beach sections, a single common set of parameters could be calculated. Some parameters may be allowed to vary in specific circumstances, for example *max *and *min *might vary to take into account that, on various beach sections, the total number of nests laid can be different. When *k *nesting beach sections are compared, the likelihood of these *k *sections analyzed with a single set of M parameters is *L*. The statistic -2 In (∏ L_i_/L) is distributed as a χ^2 ^with M-ΣM_i _degrees of freedom.

## Authors' contributions

MG initiated this project and conducted most of the analyses. PR refined some hypothesis and conducted some analyses. JPB helped with statistics and conducted some analyses. MHG refined some hypotheses and headed fieldwork. RW headed fieldwork. VH, EG and SC helped collect field data.

## Figures and Tables

**Table 1:** Model selection for leatherback nest counts on Ya:lima:po beach in 2002. Selected models are in bold.

Models rank by AIC	-Ln L	Parameters	AIC	Akaike weights		Nests & SD
A: No sinusoid				Intra	Inter	

S_1 _P_1 _S_2 _P_2 _K_2 _Max Min	471.31	10	962.63	0.3393		
S_1 _P_1 _K_1 _S_2 _P_2 _Max Min	471.61	10	963.22	0.2525		
**S**_**1 **_**P**_**1 **_**S**_**2 **_**P**_**2 **_**Max Min**	**472.60**	**9**	**963.22**	**0.2520**	0.0000	4652 SD 73
S_1 _P_1 _K_1 _S_2 _P_2 _K_2 _Max Min	471.16	11	964.32	0.1454		
S_1 _P_1 _S_2 _P_2 _Max	477.79	8	971.57	0.0039		
S_1 _P_1 _K_1 _S_2 _P_2 _Max	476.80	9	971.61	0.0038		
S_1 _P_1 _S_2 _P_2 _K_2 _Max	477.59	9	973.18	0.0017		
S_1 _P_1 _K_1 _S_2 _P_2 _K_2 _Max	476.79	10	973.58	0.0014		

B: One sinusoid						

**Δ**_**1 **_**φ**_**1 **_**β**_**1**_	**458.32**	**12**	**940.65**	**0.3413**		
Δ_1 _φ_1 _α_1 _β_1_	457.52	13	941.05	0.2793	0.0005	4719 SD 62
Δ_1 _φ_1 _τ_1 _β_1_	457.57	13	941.14	0.2674		
Δ_1 _φ_1_τ_1 _α_1 _β_1_	457.45	14	942.90	0.1105		
Δ_1 _φ_1 _α_1_	463.76	12	951.52	0.0015		

C: Two sinusoids						

**Δ**_**2 **_**φ**_**2 **_**α**_**2 **_**β**_**2**_	**449.21**	**16**	**930.54**	**0.6065**	**0.0864**	**4716 SD 58**
Δ_2 _φ_2 _τ_2 _α_2 _β_2_	449.07	17	932.14	0.2730		
Δ_2 _φ_2 _β_2_	452.84	15	935.69	0.0463		
Δ_2 _φ_2 _α_2_	452.93	15	935.87	0.0423		
Δ_2 _φ_2 _τ_2 _β_2_	452.21	16	936.42	0.0320		

D: Three sinusoids						

Δ_3 _φ_3 _α_3 _β_3_	441.91	20	923.822	0.3994		
**Δ**_**3 **_**φ**_**3 **_**β**_**3**_	**442.95**	**19**	**923.900**	**0.3842**	0.9132	
Δ_3 _φ_3 _τ_3 _α_3 _β_3_	441.91	21	925.822	0.1469		
Δ_3 _φ_3 _τ_3 _β_3_	444.27	20	928.550	0.0376		
Δ_3 _φ_3 _α_3_	445.44	19	928.878	0.0319		

**Table 2:** Model selection for leatherback nest counts on Ya:lima:po beach in 1987. Selected models are in bold.

Model ranked by AIC	-Ln L	Parameters	AIC	Akaike	Weights	Nests & SD
A: No sinusoid				Intra	Inter	

S_1 _P_1 _K_1 _S_2 _P_2 _Max Min	857.26	10	1734.53	0.3551		
**S**_**1 **_**P**_**1 **_**S**_**2 **_**P**_**2 **_**K**_**2 **_**Max**	**858.92**	**9**	**1735.84**	**0.1847**	0.0000	33563 SD 44
S_1 _P_1 _K_1 _S_2 _P_2 _Max	859.20	9	1736.41	0.1392		
S_1 _P_1 _K_1 _S_2 _P_2 _K_2 _Max Min	857.26	11	1736.54	0.1306		
S1 P1 K1 S2 P2 K2 Max	858.56	10	1737.13	0.0972		
S_1 _P_1 _S_2 _P_2 _K_2 _Max Min	858.94	10	1737.90	0.0661		
S_1 _P_1 _S_2 _P_2 _Max Min	860.92	9	1739.85	0.0249		
S_1 _P_1 _S_2 _P_2 _Max	864.34	8	1744.69	0.0022		

B: One sinusoid						
Δ_1 _φ_1 _τ_1 _α_1 _β_1_	844.89	14	1717.79	0.4755		
**Δ**_**1 **_**φ**_**1 **_**α**_**1 **_**β**_**1**_	**845.94**	**13**	**1717.90**	**0.4494**	0.0000	33624 SD 49
Δ_1 _φ_1 _α_1_	848.78	12	1721.56	0.0722		
Δ_1 _φ_1 _β_1_	852.30	12	1728.61	0.0021		
Δ_1 _φ_1_τ_1 _β_1_	852.32	13	1730.64	0.0008		

C: Two sinusoids						

Δ_2 _φ_2 _τ_2 _α_2 _β_2_	828.43	18	1692.86	0.5988		
**Δ**_**2 **_**φ**_**2 **_**α**_**2 **_**β**_**2**_	**829.83**	**17**	**1693.67**	**0.3992**	**0.1168**	**33424 SD 40**
Δ_2 _φ_2 _α_2_	836.10	16	1704.20	0.0021		
Δ_2 _φ_2 _τ_2 _β_2_	841.28	17	1716.56	0.0000		
Δ_2 _φ_2 _β_2_	842.35	16	1716.71	0.0000		

D: Three sinusoids						

Δ_3 _φ_3 _τ_3 _β_3_	**823.81**	**21**	**1689.63**	**0.4555**	0.8832	
Δ_3 _φ_3 _α_3 _β_3_	824.47	21	1690.94	0.2370		
Δ_3 _φ_3 _τ_3 _α_3 _β_3_	823.80	22	1691.60	0.1704		
Δ_3 _φ_3 _τ_3 _β_3_	826.21	20	1692.42	0.1129		
Δ_3 _φ_3 _α_3_	827.74	20	1695.49	0.0243		
